# Socioeconomic Status and Survival of People with Human Immunodeficiency Virus Infection before and after the Introduction of Highly Active Antiretroviral Therapy: A Systematic Literature Review

**DOI:** 10.4172/2155-6113.1000163

**Published:** 2012-07-20

**Authors:** Elena Pavlova-McCalla, Mary Jo Trepka, Gilbert Ramirez, Theophile Niyonsenga

**Affiliations:** 1 Department of Epidemiology and Biostatistics, Robert Stempel College of Public Health and Social Work, Florida International University, Florida, USA; 2 Department of Health Policy and Management, Robert Stempel College of Public Health and Social Work, Florida International University, Florida, USA

**Keywords:** Human Immunodeficiency Virus, Acquired Immunodeficiency Syndrome, Socioeconomic status, Systematic review, Mortality

## Abstract

**Background:**

Human immunodeficiency virus/acquired immunodeficiency disease syndrome-associated mortality contributes considerably to overall mortality rates among adults in the United States. The purpose of this review is to systematically examine conceptual approaches that have been used to evaluate the association between socioeconomic status of people infected with human immunodeficiency virus and their survival and summarize existing evidence regarding the association between socioeconomic status and mortality due to human immunodeficiency virus/acquired immunodeficiency disease syndrome.

**Methods:**

We systematically retrieved neighborhood and individual-level studies of acquired immunodeficiency disease syndrome-related or all-cause mortality among patients diagnosed with human immunodeficiency virus that reported original data and analyzed socioeconomic status as a predictor of mortality.

**Results:**

We included 21 studies (19 cohort and 2 case-control studies). Heterogeneity in both the conceptual approaches to socioeconomic status measurements and selection of variables for the adjustment of the measure of association precluded meta-analysis of the results. Six studies observing populations before the introduction of highly active antiretroviral therapy found that socioeconomic status was not associated with human immunodeficiency virus/acquired immunodeficiency disease syndrome mortality. In the post- highly active antiretroviral therapy period socioeconomic status was inconsistently associated with Human immunodeficiency virus/acquired immunodeficiency disease syndrome mortality risk in studies adjusting for highly active antiretroviral therapy use.

**Conclusion:**

Further studies considering multilevel socioeconomic status measurements and controlling for treatment and clinical variables are needed to enhance understanding of the role of socioeconomic gradients on human immunodeficiency virus outcomes.

## Introduction

Human immunodeficiency virus/acquired immunodeficiency syndrome (HIV/AIDS) remains an important public health problem despite significant worldwide efforts to combat the disease. The availability of highly active antiretroviral therapy (HAART) in the United States (US) and other developed countries altered the progression of HIV infection and brought about a dramatic decline in mortality due to HIV/AIDS [1–4], increased the median age at death due to HIV [5], and improved life expectancy after HIV diagnosis [6]. However, the rates of decline in HIV/AIDS morbidity and mortality have flattened in recent years among all racial/ethnic groups [7,8]. By the end of 2009, an estimated 1,108,611 people developed AIDS in the US, and of those, over half have died [9]. Even though survival has increased markedly, HIV/AIDS continues to be one of the major causes of premature mortality in the US and young minority populations have been particularly affected. In 2007, it was the fourth leading cause of death for non-Hispanic black (NHB) males and third for NHB females aged 25–44 years, ranking higher than their respective counterparts in other racial/ethnic groups (CDC, 2010) [10–12]. Therefore, for certain groups, HIV-related mortality continues to be a particularly acute public health problem.

Early HIV testing [13–16], access to specialized care [17], and adherence to HAART [18–20] are critical for control of disease progression. Yet, explanation of evident racial/ethnic disparities in HIV/AIDS patterns requires looking at complex characteristics. Population mobility, migration, and urbanization modify interactions between susceptible and infected persons in populations [21]. In addition, broad challenges involving economic and social deprivation, socialization patterns, socially inflicted trauma, targeted marketing of illicit drugs, and limited access to health care may explain differences in health outcomes [22]. The facts that are nearly 1 in 4 NHBs live in poverty in the US and that the poverty rate for NHBs remains about three times that of non-Hispanic whites (NHW) may partly explain the racial/ethnic variations in the national rates of HIV/AIDS incidence [23–25]. Studies observing associations between socioeconomic status and incidence of HIV/AIDS have illustrated that problems related to poverty, including limited access to quality care; the exchange of sex for drugs, money, or to meet other needs; and increased levels of substance use can directly or indirectly increase HIV risk factors [26,27]. Queries whether the social and economic situations of HIV-infected individuals explain variations in the disease progression and survival are supported by the framework of social production of disease and political economy of health. According to this framework, a person’s relative social and economic positioning shapes behavior, and the relationship between subordinate-dominant groups affects patterns of disease through material and social inequalities [28].

To date, research examining the role of SES on HIV/AIDS mortality has been scarce. In addition to the difficulty of obtaining estimates of individual-level socioeconomic measures, the limited research could be due to measurement problems arising from the lack of conceptual clarity about the essential nature of social stratification and the absence of the application of sound measurement theory to the construction of socioeconomic measures in relation to health outcomes [29]. Socioeconomic status (SES) describing a person’s position in a society is a multidimensional construct [29], but due to limited resources, most health studies measure SES using a single socioeconomic variable measured at a single time point and level [30]. This may be particularly restrictive in studies of HIV infection because socioeconomic factors affecting health of HIV-infected patients change not only at different times in the life course [31], but also in the course of the disease through different causal pathways [32,33].

The present review aims to explore the conceptual approaches that have been used to examine associations between SES of individuals diagnosed with HIV/AIDS and their survival and to systematically evaluate the existing evidence regarding the association between socioeconomic gradients and patterns of HIV/AIDS mortality. Knowledge about the role of SES on premature mortality of individuals with HIV/AIDS will facilitate the tailoring of prevention programs to the needs of individuals and communities.

## Materials and Methods

A comprehensive electronic search of published materials was conducted to identify conceptual and empirical studies describing associations between the measures commonly accepted in public health for evaluation of SES and survival of persons diagnosed with HIV/AIDS. PubMed, National Library of Medicine Gateway of National Institutes of Health (NLM), Social Science Citation Index, Wilson Web-Humanities and Social Sciences Index Retrospective, FRANCIS (International Humanities and Social Studies), PsycINFO, and CINAHL Plus were searched for abstracts and full text articles published in English and other languages using the following combinations of keywords:’ HIV/AIDS, socio-economic status, survival’, ‘HIV/AIDS, survival, SES’, ‘HIV/AIDS, mortality, socioeconomic^★^’, ‘HIV/AIDS, mortality, socioeconomic index’, ‘HIV/AIDS, mortality, socioeconomic deprivation’, ‘HIV/AIDS, mortality, socioeconomic position’, ‘HIV/AIDS, mortality, economic status’, ‘HIV, mortality, neighborhood’, ‘HIV, mortality, education’, ‘HIV, mortality, income’, ‘HIV, mortality, social class’, ‘HIV, disease course^★^, socioeconomic’, ‘HIV, mortality, deprivation index.’ Papers in English, Spanish, Italian, and French were examined. In addition, the references of the retrieved publications were reviewed as part of the search.

We included studies that met the following criteria: 1) measured AIDS-related deaths or all-cause mortality among individuals infected with HIV; 2) reported original data; and 3) analyzed SES as a predictor of mortality. The exclusion criteria were studies evaluating effects of socio-economic and socio-cultural attributes on HIV/AIDS outcomes other than AIDS-related or all-cause mortality, access to care, quality of life among HIV-infected individuals, sexual behaviors, practices, and beliefs about HIV. Studies that documented non-HIV/AIDSrelated deaths and accidental deaths among HIV-infected patients were excluded from the review.

Retrieved publications were stratified by the study observation period because the introduction of the effective therapeutic intervention of HAART in 1996 in the US, Canada, Europe, and Latin American countries [4,34,35]. For the reports that studied cohorts until 1996, materials were considered as covering pre-HAART period (before introduction of HAART), and for the evaluations where subjects were followed after 1996 published research was assessed as encompassing post-HAART period (when HAART became widely available). Further, we abstracted the key concepts used in the studies for SES assessments and listed them according to the level (i.e. individual vs. community) of data collection (Table1). We selected adjusted measures of association for the studies that reported both crude and adjusted measures.

Using statistical software STATA version 10 (StataCorp LP, College Station, Texas), we estimated the magnitude of the overall heterogeneity of the studies and the index of heterogeneity after stratifying studies along various lines. We applied the methodology used by other investigators [36] to retain only the hazard ratios (HRs) and relative risks (RRs) comparing lowest and highest categories when SES variables were defined in terms of quintile or levels (e.g., level I,II,III,IV). If there was more than one socioeconomic variable, we first analyzed socio-economic resource (SER) index or socio-economic index (SEI), if present, because these are composite measures. If information on the index was not available, income and poverty status were considered next and then employment status, occupation, and education level. Income was chosen over education if both present, because income is a realized resource and education allows potential access to the desired resources [29]. However, studies of post-HAART period evaluating effects of higher SES versus low (when different SES determinants were combined) showed that the variation in effect size was 84.1 percent. The assessment of studies for pre-HAART period examining effects of lower SES vs. high with combination of SES indicators produced similar findings. Stratifications on a particular variable of SES (such as income, education level, SEI) were performed, and these too demonstrated a statistically significant high level of heterogeneity. After multiple stratifications, it became apparent that the degree of heterogeneity among studies was too large to be explained and, therefore, a decision was made that meta-analysis would not be conducted.

### Literature Search

**Figure 1:**
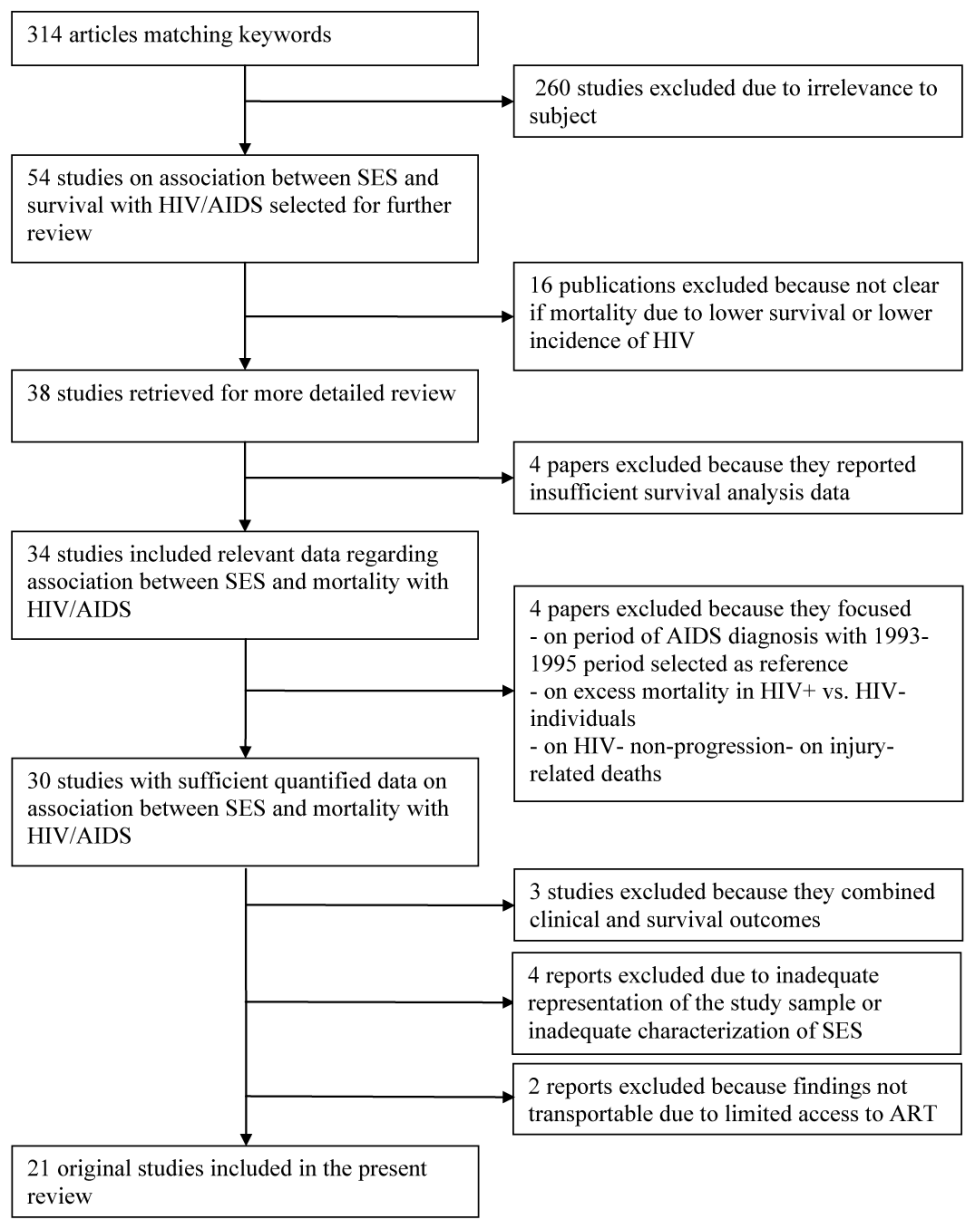
Study selection process in a literature review of the relation between SES and survival of individuals with HIV/AIDS.

Of the 314 studies found using the keywords, 54 were deemed to be relevant (Figure 1). Eight cross-sectional [37–44] and eight ecologic studies [45–52] were excluded from the analysis because they examined absolute and relative differences in mortality risk for HIV/AIDS according to the socioeconomic characteristics of the area of patients’ residence at the time of AIDS diagnosis. Therefore, it was not possible to determine the extent of mortality due to poor survival of people with HIV/AIDS versus differences in HIV incidence and prevalence by socioeconomic group. Four publications were not considered for the analysis because they provided insufficient survival analysis data [53–56].

One study was excluded because in comparison of survival probability among persons with AIDS (PWA) at 24 months after AIDS diagnosis significant differences in the AIDS incidence for low and high income levels were not adequately addressed [57]. This study also reported mortality risk for the 2-year periods before and after introduction of HAART with 1993–1995 period selected as reference mainly showing effects of HAART rather than magnitude of the problem in relation to SES. Another was excluded because the excess mortality in HIV-positive patients according to socio-demographic characteristics was reported in relation to a HIV-negative cohort [58]. A Swiss study was excluded because it evaluated social co-factors of HIV-non-progression but not mortality [59]. A cohort study on mortality among HIV-infected participants in the Women’s Interagency HIV Study and the Multicenter AIDS Cohort Study were excluded because it focused only on accident- and injury-related deaths [60].

Three reports were eliminated because they combined death with clinical outcomes (e.g., all-cause hospitalization and death/clinical progression) so it was not possible to separate the effect of SES on death from other outcomes [61–63]. One report was excluded because of concern that the outcome of hospital mortality and the measure of SES type of admission as a proxy for individual SES would limit the generalizability of findings [64]. Concerns regarding non-representativeness of the study sample lead us to exclude one US study that explored relationships between crack use and HIV disease outcomes among women while evaluating income and education as potential confounders [65]. Similarly, a Canadian report examining effects of self-reported income on mortality among HIV-infected MSM and a Spanish study evaluating role of education on mortality risk among HIV-infected IDUs were removed from this review due to limitations involving the cohorts with one transmission mode [66,67].

Two studies done in African countries were excluded because of the generalizability concerns arising when results are compared between “high” and “low” income countries and when there is inadequate information on differentials regarding definitions of SES and modifying effects of countries’ social contexts characterized by high levels of economic inequality and limited access to HAART. The first of these studies, conducted in South Africa, compared the hazards of all-cause death in treatment-naïve HIV-infected adults initiating ART and having some monthly income versus no monthly income during one-year follow-up [68]. It was not clear if the mortality was related to the earned income/government disability grants or continuous access to health care and the disease stage when patients became eligible for disability grants. The second study, carried out in Rwanda, was limited to only ART-naïve HIV-positive women of childbearing age and found low household income as a statistically significant independent predictor of mortality [69]. Thus, 21 publications of the period between 1992 and 2009 were included in the present review (Table 1).

## Results

Of the 21 studies included in this review, 14 originated from North America and seven from Europe (Table 1). Four studies examined effects of SES on mortality with HIV/AIDS in the years of pre-HAART period [70–73]. Fifteen studies provided an assessment of SES in relation to survival in HIV-infected patients during the years of commercial availability of HAART [74–88]. Two studies [34,35] evaluated the impact of SES on survival with HIV/AIDS in both periods. Investigators used either patients’ self-report of the socioeconomic determinants or linkage of the individuals’ residence at the time of diagnosis with the census data. Understandably, cost and difficulties in obtaining individual SES data cause researchers’ reliance on census data as a surrogate for SES assessment. However, studies with the area-level of SES often failed to specify their conceptual approaches as to whether the census data were applied as a proxy of individual SES or as an assessment of contextual socioeconomic effect. There were no studies with multilevel SES measurements.

While efforts to operationalize SES continue to evolve, current SES assessment in relation to health generally relies on data from occupational position, education, income, poverty status, or any combination of these measures [29]. The present review of studies on the relationship between SES and mortality risk among patients with HIV/AIDS confirmed the consistency of this approach. Most of the studies included in the review used more than one of the common determinants to measure SES. Nine reports [34,35,71,75,76,80,81,84,85] presented one arbitrarily selected measure to quantify the association between SES and mortality among people with HIV/AIDS. Education was the most frequently used measure of SES followed by income, and employment status (Table 1). The most common rationale for the selection of a particular socioeconomic measure in the reports was that it had been adopted in the past and had high predictive validity. A calculated SES index was used in five studies [34,71,79,82,88] because of a perception that a complex index discriminates better than its components. Homelessness and access to health insurance or the type of health insurance as a factor affecting survival with HIV/AIDS was considered only in the US studies of post-HAART period indicating the importance of these variables in the pathways between effective therapy initiation and survival.

Studies also differ in terms of studied population. Seven reports provided data on survival of persons with AIDS (PWAs) only [34,71,72,79,80,85,88], one examined survival of HIV-infected parents of adolescents [73], one looked at survival in HIV-infected children [78], and the remaining studies assessed HIV-infected patients of both genders and various age groups (Table 1). While the majority of retrieved publications looked at all-cause mortality in individuals diagnosed with HIV/AIDS, two Canadian reports [75,83] and one US study [80] covering the post-HAART period described HIV/AIDS-related mortality. Crude HR or RR of death was reported in eight studies and adjusted measures of association were found in the remaining (Table1).

### Studies of Pre-HAART Period

Four retrieved studies of pre-HAART period evaluated the risk for mortality in relation to SES in samples of individuals with the range of mean ages from 22 to 39 years and the range of median follow-up from 16.1 months to 28 months [70–73]. Significant association between education and mortality in HIV-infected patients was found only in one US study [73] which found that adults without a high school diploma were at a higher risk of death. The same study found that perceived financial status (comfortable vs. very poor) was not associated with mortality, but availability of social support significantly decreased risk of death in the cohort of parents with adolescent children. While education partly serves as an indicator of social capital, the key factor of material capital, income, did not affect mortality in a large population of HIV-infected patients with different transmission modes [70]. However, in the same study, an indicator of human capital, employment status at baseline, was a statistically significant protective factor for mortality with HIV/AIDS in crude analysis [70].

One Italian study calculated a composite socioeconomic measure including variables of income and social support and did not find statistically significant effects of socioeconomic resource index (SER) on mortality among PWA [71]. These results were consistent with the findings of the only study of the pre-HAART period that adjusted the measure of association for demographic and clinical variables. Using census block group-level socioeconomic data, this study did not find statistically significant association between mortality in persons diagnosed with AIDS and living in a neighborhood characterized by poverty, working class occupation, or low educational level [72].

### Studies of post-HAART period

Included in the review, 15 reports of post-HAART period examined relationships between SES and all-cause mortality and HIV/AIDS-related mortality among HIV-infected patients with the mean age ranging from 11 to 42.5 years and the median follow-up ranging from 12 months to 8.6 years [74–88]. The composite index of one of these studies was based on the variables of availability of stable income, owned residential property, the need to receive social support, and the effective availability of this support in the past and at the time of interview [82]. Measured by this index, low SES significantly increased mortality risk of individuals diagnosed with HIV after adjustments for clinical variables [82]. In contrast, SES measured at area-level by a composite index was not associated with increased mortality in PWAs after adjustments for demographic and clinical variables, stage of the disease, and ART initiation [79,88].

Low household income was a significant predictor of mortality among patients with HIV/AIDS in three Canadian studies, a setting with universal access to health care [75,83,86]. However, one study with individually collected SES data did not find an association between income and HIV/AIDS mortality risk [77]. The lack of such association was supported in two reports that applied area-level of SES data: a US study evaluating unadjusted risk of death in HIV-infected patients [87] and an Italian study examining mortality among PWA after adjustments for relevant variables [79]. Only one US study assessed wealth. Selfreported lack of accumulated assets in the population-based sample of people with HIV/AIDS was associated with statistically significant increased risk for all-cause and HIV-related mortality even after adjustments for sociodemographic, clinical, and treatment variables [77].

Five studies carried out in different countries indicated that education beyond a high school degree was a statistically significant protective factor for all-cause and HIV/AIDS-related mortality [77,78,82,83,86], and one report showed no significant association with education [87]. Poverty as the area-level SES factor was positively associated with an increased risk of death in HIV-infected patients even after adjustments for age, clinical and treatment variables [83,84]. The only study that examined the effect of social context on survival of HIV-infected children found that maternal unemployment was associated with lower survival after adjustment for baseline AIDS diagnosis [78]. Lack of employment, an important dimension of maternal, social, and human capital, was found to be associated with an increased risk for HIV/AIDS mortality in four other studies applying this measure after adjusting for demographic, clinical and treatments factors [77,81,83,86].

The effects of social capital indicators were also significant. Stable partnership was a protective factor for mortality in a Swiss cohort of HIV-infected patients after adjustments for education, ART, demographic and clinical variables [76]. Living situation characterized by unmarried status and not living with a partner was a significant predictor of mortality in a case-control study [74]. The same case-control study demonstrated that homelessness increased the risk of death in patients with HIV, but the association was not assessed for potential confounders. Another US study using an adjusted measure of association between homelessness and HIV/AIDS mortality risk found no statistically significant association [77].

Six studies used type of insurance as the measure for SES. An earlier publication of the post-HAART period showed that HIV-infected patients receiving care from Medicaid were at a higher risk of death compared with those in care under provisions of Ryan White Act [74]. Lack of insurance after adjustment for sociodemographic and clinical variables was also associated with a reduced relative risk of death as compared with having private insurance in a cohort of persons diagnosed with HIV/AIDS [77]. Recent studies, however, indicated that lack of health insurance or having Medicaid or Medicare compared with private insurance increased the risk of death [80,85,87,88]. However, adjustments for the patients’ age and the disease stage were found only in two studies [80,85]. Such adjustment of the measure of association is important because Medicare eligibility is based on age or disability status and it would be expected that people receiving Medicare would be at higher risk of death due to the facts of being older or with more advanced disease.

### Studies comparing pre- and post-HAART periods

Two reports compared mortality among persons diagnosed with HIV/AIDS before and after introduction of HAART based on area-level of SES. For both periods, no association was found in a San Francisco study between neighborhood median household income and risk of mortality in HIV-infected patients after adjustment for relevant variables including use of HAART [35]. However, an Italian study assessing SES by composite index indicated that in the post-HAART period, living in a deprived area increased risk of death among PWA after adjustment for a set of variables but not HAART use [34]. In addition to the use of various dimensions of SES, the presentation of crude measures of association, and analysis of different arrays of potential confounders allowed us to conclude that interpretation and evaluation of the results of the studies included in this review largely depend on the researchers’ conceptual approach.

## Discussion

Socioeconomic factors have been examined and postulated as an explanation for unfavorable health outcomes and excess burden of chronic diseases [89,90]. However, the direction and strength of the relationship between SES and health status depend on how SES is defined, the outcome of interest, the demographic composition of the cohorts, and the geographic regions under studies [91]. Our review confirmed that heterogeneity of available research investigating relationship between SES and mortality risk among HIV-infected individuals preclude making definite conclusions whether SES is an independent predictor of mortality in people diagnosed with HIV/AIDS or a modifying factor for HIV progression to death. This is the first review evaluating studies about the association between SES and mortality among people with HIV/AIDS for the periods before and after introduction of HAART. A more uniform conceptualization of the socioeconomic status both at the individual and neighborhood levels with regards to HIV is needed to better understand the role of SES on mortality of people diagnosed with HIV/AIDS.

The results of the review revealed that six studies observing subjects’ survival in the pre-HAART period (including the two studies covering both periods) [34,35,70–73] did not find a statistically significant association between mortality risk and low income [35,70], poor financial status [73], poverty [72], or living in a neighborhood characterized by low SES [34], and higher percent of working class population [72]. While work is the major structural link between education and income or material condition, only one report of pre-HAART period demonstrated protective effects of employment at baseline for mortality with HIV/AIDS [70]. Conflicting results regarding effects of education on mortality of HIV-infected patients [70,73] supported the notion that educational achievement at the individual level does not allow to trace the relationship with health because education has different social meaning and consequences in different times and settings [92]. In addition to variations of SES measurements, the lack of the clear effect of SES on mortality risk was likely due to the limited effectiveness of HIV treatment in the pre-HAART period and thus factors linking SES to mortality through the ability to access and negotiate health care would not have greatly influenced survival.

On the other hand, the studies of post-HAART period generally showed greater all-cause mortality and HIV/AIDS-related mortality risk for people living in neighborhoods characterized by low socioeconomic index [34,82], low income [75,83,86], and high levels of poverty [84]. However, this association was not statistically significant in most of the studies where adjustments of the mortality risk were made for HAART use [35,77,88], suggesting that the observed advantage conferred by being high SES may be at least partly related to having better access to treatment. While four out of seven papers of post-HAART period did not find increased mortality risk for people with low income [55,77,79,87] four out of five studies examining effects of education showed that higher educational achievement was protective [77,82,83,86].

These findings are somewhat expected, since once more effective treatment becomes available, access to care can make a difference in survival and tends to be higher for those of higher SES. Thus, SES may exhibit modifying effects on HIV/AIDS outcomes. To bring more clarity to the role of SES on mortality with HIV/AIDS, the measures of SES should be considered in the life course perspective. All studies included in the review measured SES at one time, usually adulthood. This limits understanding about how SES at individual or neighborhood-level affects HIV progression to death when patients with HIV/AIDS are not able to work and live on disability allowance, but may have higher levels of education or social support available. Reverse causation between income, employment status, and health outcomes cannot be excluded. Furthermore, socioeconomic indicators interrelate and the difficulty of understanding what factor comes first or has larger impact on survival have been noted in other epidemiologic studies and systematic reviews [36,89]. Studies included in the present review did not take into account the hierarchical structure of socioeconomic indicators in terms of importance and impact degree in their data analysis.

The findings that employment at baseline significantly decreased mortality risk with HIV/AIDS before and after the introduction of HAART may reflect better baseline health among patients who were able to work. The importance of clinical staging in the analysis of survival with HIV/AIDS cannot be overstressed. People are diagnosed at different times of their disease course and socioeconomic gradients may serve as markers of the disease staging. The evidence that even in universal health care settings unemployment at baseline was highly predictive for delayed access to treatment [83] may indicate patients’ inability to work due to poor health at the time of presentation for HIV care. The fact that having private insurance decreased mortality in HIV-infected individuals [80,85–88] can be a marker of better health and an earlier stage of the disease when people are able to work and thus are eligible for private insurance, and have not exhausted their financial resources or insurance limits.

Fifteen of the 21 studies adjusted for one or more clinical variables at the time of diagnosis. AIDS diagnosis and CD4 cell counts point out HIV progression and, therefore, they should always be adjusted for in the survival analysis if available. In the post-HAART period, treatment variables, such as access to care and HAART adherence [18,93,94] affecting disease progression must be considered along with clinical variables in relation to the patients’ SES. The recent trend of an increasing proportion of older adults with more advanced HIV disease compared to younger individuals at first presentation for care [13,16] also highlights the importance of adjusting measures of association for age and AIDS diagnosis in the analysis of the effects of SES on mortality and morbidity due to HIV/AIDS.

There is also a relationship between mode of HIV transmission and risk of HIV progression to AIDS and death. Persons with different risk factors (e.g. injection drug use) may have therapy adherence problems due to their behavioral and decision-making characteristics that may have grounds in low education, low incomes, unemployment, and low occupational status [18]. It has been documented that the relative survival estimates by transmission category are significantly better for males exposed through male-to-male sexual contact as compared with other transmission groups [84]. In MSM, the disease progression may take longer time as compared with IDUs [95]. However, other studies did not confirm these findings [72,96]. HIV-infected patients who stop injecting drugs can have a reduced risk of disease progression when compared with current users [73]. In the three papers [34,35,79] that included demographic information about their studied population by the transmission factor, MSM transmission mode was associated with a higher SES level while IDU was associated with a lower SES level. Thus, differentials of mortality according to SES levels may have been due to variations of disease progression in persons with different HIV risk factors.

Most of the studies measuring SES at the community level did not explicitly state if they considered the community level SES as proxies for individual SES or as measures of the social setting quality in which the HIV-infected person lives. This is important because designing intervention programs often involves targeting specific populations or addressing community needs. For instance, in the cases of employment status measured at the individual level, unemployment may mean that the person has no marketable skills, is too sick to work, or work is not available in his/her community. In contrast, unemployment on the area-based level can indicate a loss of social support in the community due to a difficult economic situation. Ideally, multilevel studies, which measure subjects’ SES at individual and community level, should be conducted to elucidate the pathways between social determinants and HIV survival. At a minimum, reporting results of studies that use area-based SES variables should explicitly state whether the measurements are meant to be proxies for individual SES level or measures of the social environment in which the individual lives [31].

Although there is a clear need for the development and validation of a measure of SES that is appropriate for studying HIV survival, the available evidence suggests that SES is not consistently and strongly associated with all-cause and disease-specific mortality risk of HIV-infected patients when access to antiretroviral therapy is considered. The synthesis of the available up-to-date knowledge on the association between SES and mortality risk among people with HIV/AIDS indicates that SES affects patients’ survival to a different extent in various population groups at distinctive points of disease progression. The effects of SES on mortality due to HIV/AIDS are also likely through modifying other factors relating to disease progression, especially in the post-HAART period. Further population-based studies with clearer conceptualization of SES and preferably multilevel measurements are needed to enhance understanding of the role of socioeconomic gradients on HIV outcomes. Improved understanding of the relationship between socioeconomic disadvantages and outcomes of such life-threatening infection will provide necessary information for designing and tailoring of public health programs for specific population groups at higher risk for HIV progression and address the effects of SES on health in order to reduce mortality and avert potential years of life lost.

## Figures and Tables

**Table 1:** Characteristics of the selected studies.

Study name and year of publication	Country of study	Study design	Period of observation	Population sample	Source of SES data	Measure of association	SES variable	Reported value of the association measure

Pre-HAART period								
1. Chaisson et al. 1995 [70]	USA	Prospective cohort	1989–1994	1372 HIV-infected patients	Self-report	Crude RR	Income	0.98 (0.78–1.22)
							Employment	0.77 (0.67–0.89)*
							Education	1.15 (0.92–1.43)

2. Palombi et al. 1997 [71]	Italy	Prospective cohort	1992–1994	168 PWA	Self-report	Crude RR	SER index	1.02 (p-value=.8)

3. Katz et al. 1998[72]	USA	Retrospective cohort	1985–1995	18,167 PWA	Census block group	HR adjusted for gender, age, ethnicity, risk group, site & period of AIDS diagnosis, clinical variables	Poverty	1.03 (0.97–1.08)
							Working class	1.03 (0.98–1.08)
							Education	0.96 (0.9–1.01)

4. Lee & Rotheram-Borus 2001 [73]	USA	Prospective cohort	1993–1995	307 HIV-infected parents of adolescent children	Self-report	Crude RR	Education	1.43 (1.01–2.07)*
							Financial status	1.51 (0.90–2.54)
							Social support	0.77 (0.64–0.93)*

Studies comparing pre-HAART and post-HAART periods								
5. Rapiti et al. 2000 [74]	Italy	Retrospective cohort	1993–1995 & 1996–1998	1,474 PWA	Census block	HR adjusted for age, gender, CD4 count at diagnosis, risk factor, AIDS-defining illness, center of diagnosis	SES index	1.08 (0.83–1.38) (1993–1995) 2.67 (1.28–5.6)* (1996–1998)

6. McFarland et al. 2003 [35]	USA	Retrospective cohort	1980–1995 & 1996–2001	2918 HIV-infected patients	Census block	HR adjusted for age, CD 4 count, IDU status, HAART	Income	0.99 (0.98–1.00) (1980–1995) 0.93 (0.86–1.01) (1996–2001)

Post-HAART period								
7. Lieb et al. 2002 [74]	USA	Retrospective case-control	1999	120 HIV-infected cases who died 240 HIV-infected controls alive	Self-report	Crude OR	Living situation	2.70 (1.45–5.02)*
							Homelessness	2.93 (1.20–7.15)*
							Insurance (Medicaid vs. Ryan White)	3.09 (1.75–5.46)*

8. Wood et al. 2002 [75]	Canada	Prospective cohort	1996–2000	1408 HIV-infected	Census track	RR adjusted for age, ART adherence, history of IDU, CD4 count, viral load, baseline AIDS	Income	2.03 (1.32–3.12)*

9. Young et al. 2004 [76]	Switzerland	Prospective cohort	2002	3736 HIV-infected patients	Self-report	HR adjusted for age, sex, transmission group, viral load, CD4 count, disease stage, ART, education.	Stable partnership	0.59 (0.44–0.79)*

10. Cunningham et al. 2005 [77]	USA	Prospective cohort	1996–2000	2864 HIV-infected patients	Self-report	HR adjusted for age, gender, CD4 count, risk factor, homelessness, region of receiving care, clinical stage, ART	Wealth (none vs. $50,000)	1.81 (1.09–3.00)*
							Income ($10,000 vs. $25,000)	0.78 (0.56–1.08)
							Education (no high school degree)	1.52 (1.14–2.02)*
							Unemployment	1.44 (1.07–1.95)*
							Insurance (none vs. private)	0.62 (0.44–0.88)*
							Homelessness	0.99 (0.7–1.4)

11. Kozinetz et al. 2005 [78]	Romania	Prospective cohort	1999–2001	333 HIV-infected children	Self-report	HR adjusted for AIDS at baseline	Education:	0.4 (0.2–0.8)*
							Father’s	0.3 (0.1–1.0)
							Mother’s Unemployment	3.6 (1.1–11.5)*

12. Palange et al. 2005 [79]	Italy	Retrospective cohort	1996–2002	1,368 PWA	Census track	HR adjusted for age, gender, risk factor, period and hospital of diagnosis, CD4 count, AIDS-defining illness	SES level	1.09 (0.77–1.54)
							Income	1.38 (0.96–1.98)

13. Jain et al. 2006 [80]	USA	Retrospective cohort	1996–2002	5007 PWA	Medical record	HR adjusted for HAART use, OI at AIDS diagnosis, age, race, risk factor	Insurance (private vs. public)	0.55 (0.5–0.6)

14. Delpierre et al. 2008 [81]	France	Prospective cohort	1996–2006	6805 HIV-infected patients	Self-report	HR adjusted for period of HIV diagnosis, medical center, age, risk factor, ART	Unemployment	3.75 (2.11–6.6)*

15. Liotta et al. 2008 [82]	Italy	Prospective cohort	1994–2005 & 1996–2005	382 HIV-infected patients 161 HIV-infected patients	Self-report	Crude RR RR adjusted for clinical staging, CD4 count	Housing	1.91 (1.15–3.17)*
							Education	0.71 (0.58–0.87)*
							SEI	4.14 (1.28–13.4)*

16. Joy et al. 2008 [83]	Canada	Prospective cohort	1997–2005	2168 HIV-infected patients	Census track	Crude HR HR adjusted for age, CD4 count, viral load, adherence, late access to ART	Unemployment	1.53 (1.28–1.78)*
							Income	0.95 (0.94–0.97)*
							Poverty	1.07 (1.01–1.13)*
							Education	0.80 (0.71–0.91)*

17. McDavid-Harrison et al. 2008 [84]	USA	Prospective-retrospective cohort	1996–2006	HIV-infected 174,569 men & 74,128 women	Census county level	RR adjusted for time from HIV diagnosis, age, race/ethnicity, risk factor, CD4 count, AIDS	Poverty:	
							for men	1.3 (1.16–1.47)*
							for women	1.77 (1.43–2.20)*

18. Chen et al. 2009 [85]	USA		1996–2004	PWA 266 cases & 1173 controls	Medical records	OR adjusted for age, year of diagnosis, stage of disease, year of HAART initiation & regimen	Insurance (public vs. private)	2.80 (1.77–4.42)*

19. Druyts et al. 2009 [86]	Canada	Prospective cohort	1997–2005	533 HIV-infected patients	Census track	Crude HR	Unemployment	2.2 (1.67–2.89)*
							Education	0.6 (0.5–0.73)*
							Income	1.29 (1.17–1.42)*

20. Silverberg et al. 2009 [87]	USA	Retrospective cohort	1996–2005	4686 HIV-infected patients	Census track Medical record	Crude HR	Education	0.85 (0.62–1.15)
							Income	0.78 (0.53–1.15)
							Insurance	1.78 (1.28–2.48)*

21. Arnold et al. 2009 [88]	USA	Prospective-retrospective cohort	1996–2006	4211 PWA	Census zip code	HR adjusted for age, race/ethnicity, CD4 count, risk factor, ART initiation	Neighborhood socioeconomic context)	0.85 (0.67–1.07)
							Insurance (private vs. none	0.65 (0.52–0.8)*

* Statistically significant association

**Abbreviations:** ART: Antiretroviral Therapy; HAART: Highly Active Antiretroviral Therapy; HARS: HIV/AIDS Reporting System; IDU: Intravenous Drug User; PWA: Persons with AIDS; SEI: Socioeconomic Index; SER Index: Socioeconomic Resource Index; Adj: Adjusted; HR: Hazard Ratio; Het: Heterosexual; Lev: Level of SES; MSM: Men Who Have Sex with Men; OR: Odds Ratio; RR: Relative Risk

## Abbreviations

AIDS: Acquired Immunodeficiency Syndrome
ART: Antiretroviral Therapy
CD4: Cluster of Differentiation 4
HAART: Highly Active Antiretroviral Therapy
HIV: Human Immunodeficiency Virus
HR: Hazards Ratio
IDU: Injection Drug Use
MSM: Men Who have Sex with Men
NHB: Non-Hispanic Black
NHW: Non-Hispanic Whites
PWA: People with AIDS
RR: Relative Risk
SEI: Socio-Economic Index
SER: Socio-Economic Resource Index
SES: Socioeconomic Status
US: United States
